# Metanephrine mirage: distinguishing the phaeocopies, a case report and literature review

**DOI:** 10.1186/s40842-024-00198-1

**Published:** 2024-11-16

**Authors:** Joanna Y. Gong, Debbie Gordon, Sylvia Ye, Bill Fleming, Jason Tan, Toby Hulf, Maryam Shamassi, Lisa M. Orme, Nezor Houli, Emma Boehm, Christopher J. Yates, Dev A. Kevat

**Affiliations:** 1 Department of Endocrinology and Diabetes, Western Health, 176 Furlong Rd, St Albans, VIC 3021 Australia; 2 Department of Endocrinology and Diabetes, Eastern Health, 5 Arnold St, Box Hill, VIC 3128 Australia; 3 Department of Endocrine Surgery, Western Health, 176 Furlong Rd, St Albans, VIC 3021 Australia; 4 Department of Anatomical Pathology, Dorevitch Pathology, Western Health, Cnr Gordon St & Ballarat Rd, Footscray, VIC 3011 Australia; 5 The Children’s Cancer Centre, The Royal Children’s Hospital, 50 Flemington Rd, Parkville, VIC 3052 Australia; 6 Department of Medicine, University of Melbourne, 300 Grattan St, Parkville, VIC 3052 Australia; 7 Department of Medical Oncology, Peter MacCallum Cancer Centre, 305 Grattan St, Melbourne, VIC 3000 Australia; 8 Department of General Surgery, Western Health, Cnr Gordon St & Ballarat Rd, Footscray, VIC 3011 Australia; 9 Department of Nuclear Medicine, Peter MacCallum Cancer Centre, 305 Grattan St, Melbourne, VIC 3000 Australia; 10 Department of Endocrinology and Diabetes, Monash Health, 246 Clayton Rd, Clayton, VIC 3168 Australia

**Keywords:** Neuroblastoma, Adrenal, Metanephrine, Incidentaloma, Phaeochromocytoma, Malignancy, Catecholamine

## Abstract

**Background:**

We present one of only seven reported cases of a catecholamine-secreting adrenal neuroblastoma in an adult. The case is used as a platform to discuss key biochemical, genomic and imaging considerations that are central to the successful, targeted management of catecholamine-secreting adrenal tumours.

**Case presentation:**

A 63-year-old male was urgently reviewed at a tertiary hospital endocrinology outpatient clinic for a 12 cm right-sided adrenal incidentaloma. Plasma normetanephrine and 3-methoxytyramine levels were approximately 10 times the upper limit of normal at 9272 pmol/L (< 900) and 1023 pmol/L (< 110), respectively.

The adrenal mass appeared to be inseparable from the liver on imaging, and thus was suspected to be an invasive malignant phaeochromocytoma. FDG positron emission tomography (PET)/CT demonstrated moderate to intense metabolic activity within the right adrenal mass. [68 Ga]Ga-DOTATATE (Ga-TATE) PET-CT demonstrated patchy, heterogenous somatostatin receptor (SSTR) expression in the adrenal lesion, at most Krenning 3 (intensity above liver).

The patient underwent a right adrenalectomy and segment 6/7 liver resection. Histopathology revealed a 130 mm diameter neuroblastoma of the differentiating subtype with a low Mitosis-Karyorrhexis Index. There was lymphovascular invasion and tumour focally present at the resection margin, but no tumour in one periadrenal lymph node, and no tumour invasion in the adherent liver. Immunohistochemistry revealed ALK positivity (+ 3) and wild type ATRX.

At nine months following adrenalectomy, the plasma normetanephrine level has reduced to 991 pmol/L (< 900). Post-operative GaTate PET/CT shows no definite abnormal SSTR-expressing lesions in the surgical bed or elsewhere. The patient has completed adjuvant radiotherapy and is a candidate for ALK-targeted therapy if required for recurrence in the future.

**Conclusions:**

Neuroblastomas may be misdiagnosed as phaeochromocytomas given the ability to secrete catecholamines and similarities in radiological appearance. Differentiating neuroblastomas from phaeochromocytomas and paragangliomas (PPGL) is critical, but clinically difficult. Genomics are central for management; diagnosing ALK-positive neuroblastoma triggers consideration of ALK-targeted therapy, which is not relevant for PPGL. A critical eye is required for the accurate diagnosis and management of malignant adrenal incidentalomas.

## Background

Neuroblastomas are undifferentiated tumours originating from sympathetic ganglia, and may be mistaken for a phaeochromocytoma or paraganglioma (PPGL). We present the case of a catecholamine-secreting adrenal neuroblastoma in an adult, one of only seven such reported cases [1–7].

Adrenal incidentalomas are found in 2% of the population [8]. It was previously thought that up to 10–15% of adrenal incidentalomas were associated with hormone excess [8, 9]. A recent study, however, of incidentalomas greater than 1 cm in diameter found 28.2% of adrenal incidentalomas were functional [10]. Around 2% of adrenal incidentalomas are adrenal cortical cancers, and other less common tumours may present a complex and confounding clinical picture [8].

Neuroblastoma is a very rare cause of an adrenal incidentaloma in adults. While also rare, PPGLs are more likely to occur in adults and originate from the adrenal chromaffin cells and autonomic ganglia respectively. We will describe the genomic features of neuroblastomas, how this differs from PPGLs, and key genomic and imaging considerations for successful therapy.

## Case presentation

A 63-year-old male was urgently reviewed at a tertiary hospital endocrinology outpatient clinic for a 12 cm right-sided adrenal incidentaloma. This was identified on a chest computed tomography (CT) scan performed to investigate weight loss. He was a heavy smoker (59 pack-year history), with chronic obstructive pulmonary disease, schizophrenia and depression. His mental health was stable, and medications included clozapine and venlafaxine.

The patient had asymptomatic hypertension with a systolic blood pressure of 134 − 163 mmHg. He reported no palpitations or headaches. Plasma normetanephrine and 3-methoxytyramine (3-MT) levels were approximately 10 times the upper limit of normal at 9272 pmol/L (< 900) and 1023 pmol/L (< 110), respectively. Plasma metanephrine levels were unremarkable. A 24-h urine collection was concordant, with an elevated normetanephrine:creatinine ratio of 2.6 mmol/mol (< 0.25), 3-MT:creatinine ratio of 2168micromol/mol (< 197), and metanephrine:creatinine ratio of 0.17 mmol/mol (< 0.10). The aldosterone:renin ratio, 1 mg dexamethasone suppression test, serum sodium, serum potassium and DHEA-S levels were unremarkable (Table 1).

**Table 1:** Biochemistry prior to and following adrenalectomy

	**Pre-operative**	**Post-operative**	**Reference range**
**Plasma**			
Normetanephrine	9272 pmol/L	991 pmol/L	< 900 pmol/L
Metanephrine	368 pmol/L	−	< 500 pmol/L
3-methoxytyramine	1023 pmol/L	134 pmol/L	< 110 pmol/L
**24-h urine**			
Normetanephrine:Cr	2.6 mmol/mol	0.26 mmol/mol	< 0.25 mmol/mol
Metanephrine:Cr	0.17 mmol/mol	0.05 mmol/mol	< 0.10 mmol/mol
3-methoxytyramine:Cr	2168 μmol/mol	−	< 197 μmol/mol
**Other functional testing**			
1 mg DST	61 nmol/L	−	< 50 nmol/L
Salivary cortisol	2.3 nmol/L	−	3 − 35 nmol/L
24-h urine cortisol	161 nmol/L (volume 2180 mL)	−	< 280 nmol/L
Aldosterone:renin ratio	34	−	< 71
Sodium	138 mmol/L	−	135 − 145 mmol/L
Potassium	4.4 mmol/L	−	3.5 − 5.2 mmol/L
DHEA-S level	0.3 μmol/L	−	3.0 − 10.5 μmol/L

*Cr* Creatinine, *DST* Dexamethasone suppression test

**Fig. 1:**
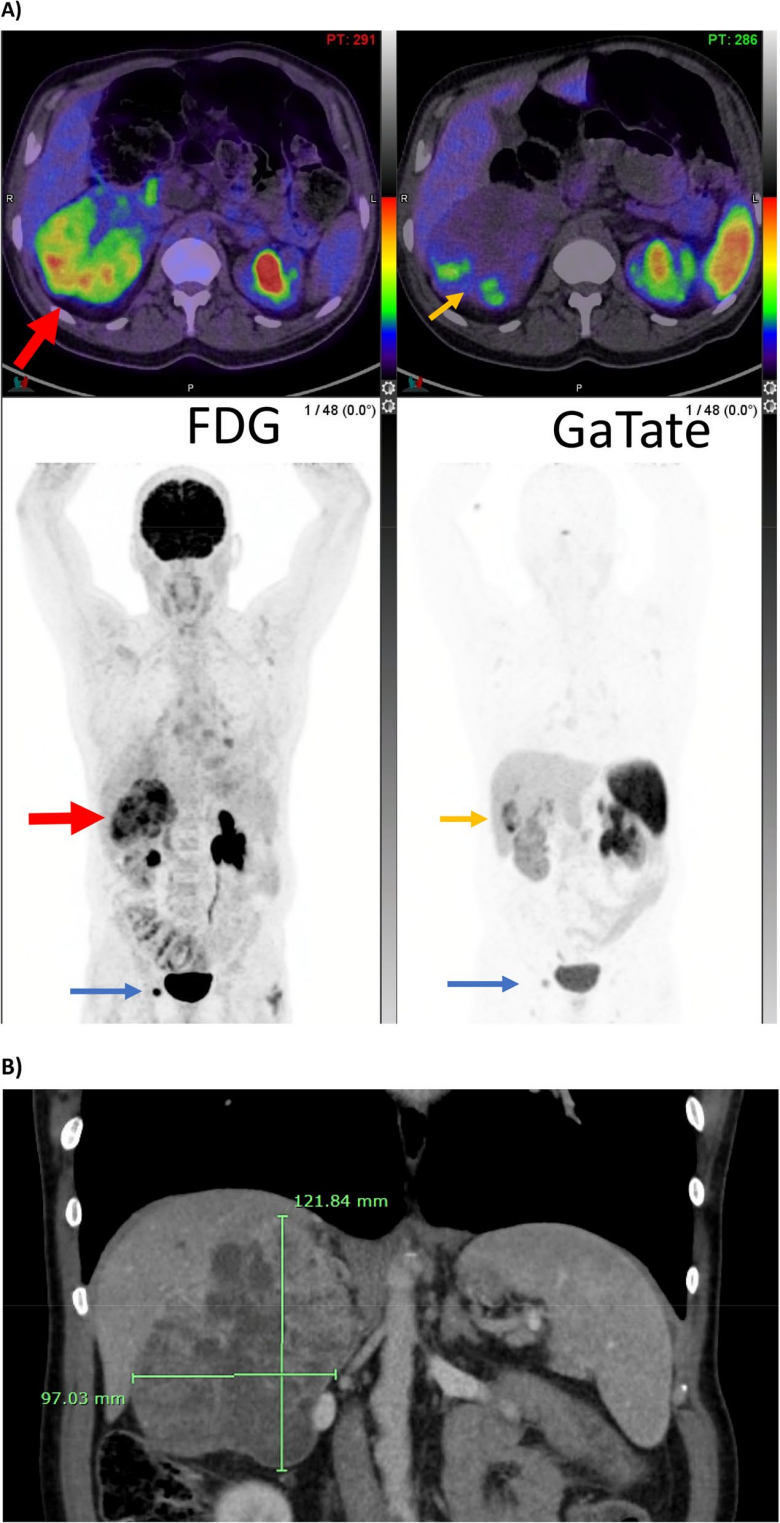
**A** FDG (left panel) and GaTate (right panel) maximum intensity projections (bottom) and upper abdominal axial slice demonstrating a moderate to intensely FDG-avid right adrenal lesion (red arrows) with corresponding patchy, peripheral somatostatin receptor expression on GaTate (orange arrows), as well as a single intensely FDG-avid right inguinal lymph node (blue arrows). **B** Contrast adrenal CT demonstrating measurements of the large right adrenal mass (rulers)

A dedicated contrast adrenal CT scan confirmed a right adrenal mass measuring 12.2 cm × 9.7 cm × 10.7 cm. There was heterogeneous post-contrast enhancement with enhancing solid areas and non-enhancing necrotic and cystic areas. It was not consistent with a lipid-rich adrenal adenoma, with low absolute and relative contrast washout estimated at 39% and 28%, respectively. The adrenal mass was thought to be inseparable from the liver on imaging (Fig. 1), and thus suspected to be an invasive malignant phaeochromocytoma. FDG positron emission tomography (PET)/CT demonstrated moderate to intense metabolic activity within the right adrenal mass and an intensely-avid right inguinal node, raising concern for metastasis. On [^68^Ga]Ga-DOTATATE (Ga-TATE) PET-CT, the adrenal lesion demonstrated patchy, heterogenous somatostatin receptor (SSTR) expression, at most Krenning 3 (intensity above liver). An echocardiogram was unremarkable.

The patient was commenced on alpha-blockade with phenoxybenzamine. It was initiated at 10 mg twice daily, then uptitrated to 30 mg twice daily to achieve a systolic blood pressure of 100-110 mmHg.

Given the patient’s comorbidities and high mortality risk with attempted surgery, a multidisciplinary team discussion recommended an excisional inguinal node biopsy to exclude metastases. The inguinal node biopsy showed reactive tissue only.

**Fig. 2:**
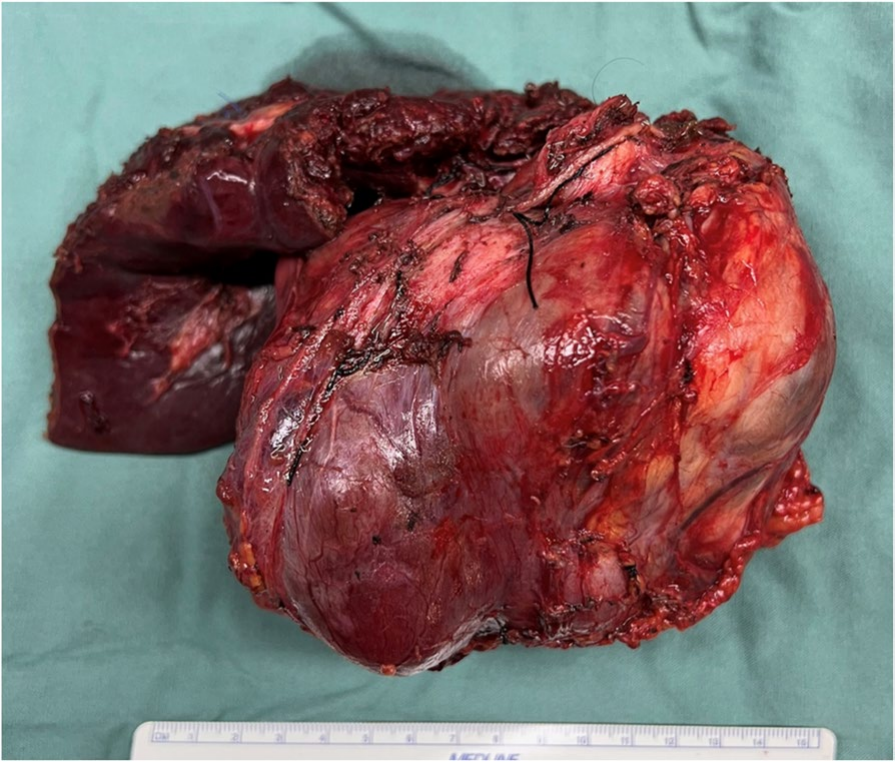
Right adrenal mass, with partial liver resection

**Fig. 3:**
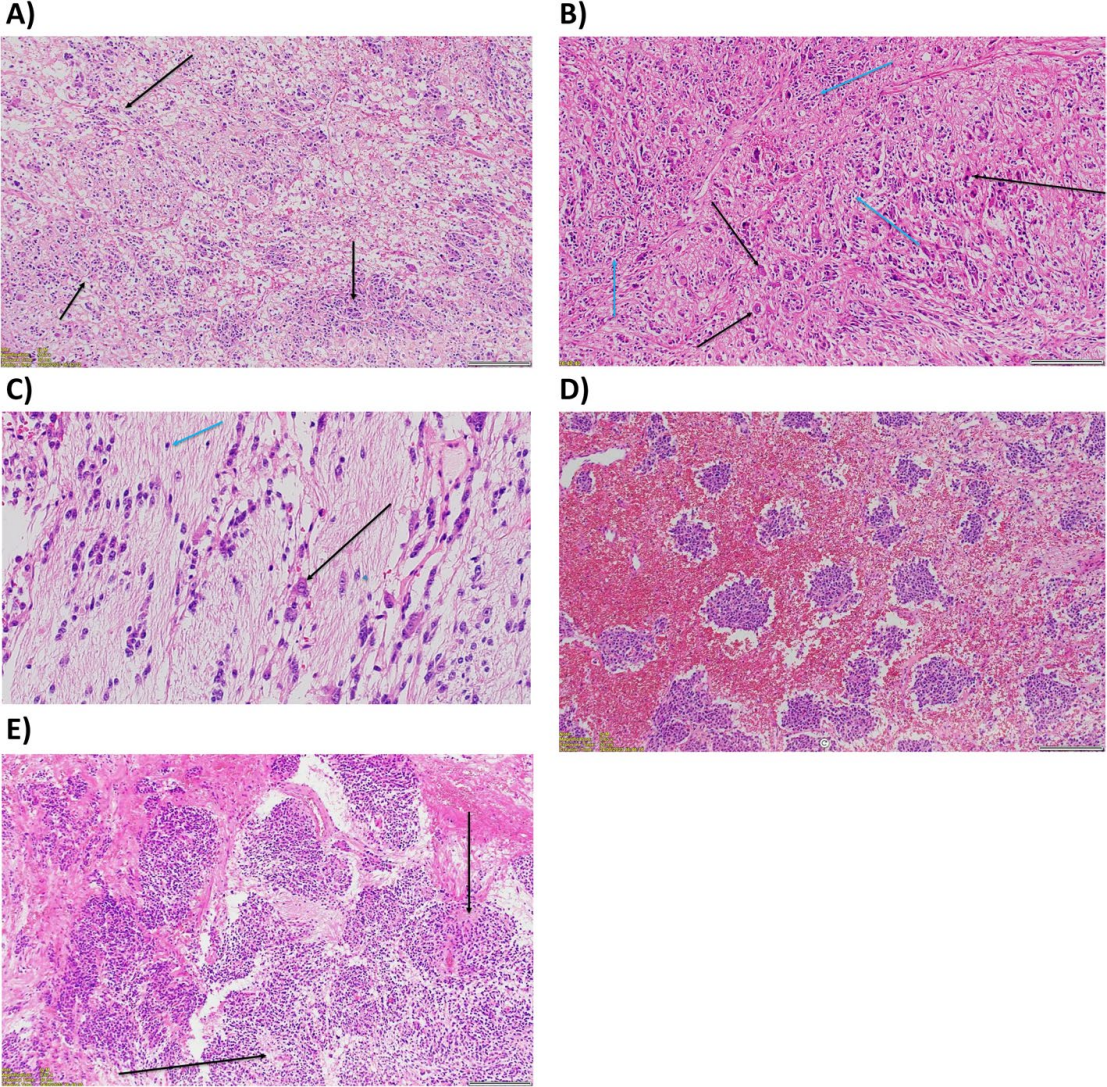
Resected neuroblastoma histology, demonstrating **A** Low-power view of the tumour with nests of small blue primitive-appearing cells floating within an oedematous and fibrillary background, **B** Medium power view demonstrating a variety of tumour cells ranging from small- and primitive-appearing (neuroblasts, yellow arrow) to larger cells with moderate amounts of eosinophilic cytoplasm and large nuclei (ganglion cells, black arrow) against a neurofibrillary background, **C** Higher power view demonstrating morphology of tumour cells in different stages of maturation from neuroblasts (blue arrow) to ganglion cells (black arrow), **D** Nests of undifferentiated neuroblasts against a haemorrhagic background, and **E** Pseudo-Rosette formation: arrangement of tumour cells around a central area filled with neurofibrillary processes

Given suspected hepatic invasion, the patient then proceeded to a right adrenalectomy and segment 6/7 liver resection (Fig. 2). Histopathology revealed a 130 mm diameter neuroblastoma of the differentiating subtype with a low Mitosis-Karyorrhexis Index. There was lymphovascular invasion and tumour focally present at the resection margin, but no tumour in one periadrenal lymph node and no tumour invasion in the adherent liver (Fig. 3). Immunohistochemistry revealed ALK positivity (+ 3) and wild type ATRX.

Post-operatively, phenoxybenzamine was ceased, and he recovered well after a brief period of inotropic support. At nine months following adrenalectomy, the plasma normetanephrine level has reduced to 991 pmol/L (< 900), with persistent minor elevation attributed to venlafaxine. The 24-h urine normetanephrine:creatinine ratio is 0.26 mmol/mol (< 0.25), and the metanephrine:creatinine ratio is 0.05 mmol/mol (< 0.10). Post-operative GaTate PET/CT shows no definite abnormal SSTR-expressing lesions in the surgical bed or elsewhere. The patient has completed adjuvant radiotherapy and is a candidate for ALK-targeted therapy if required for recurrent or metastatic disease in the future.

## Discussion and conclusions

Neuroblastic tumours arise from sympathetic ganglion cells, comprising of neuroblastomas (immature, malignant), ganglioneuroblastomas (intermediate), and ganglioneuromas (mature). Neuroblastomas usually affect children, being extremely rare in adults. The most common sites for adult-onset neuroblastoma are adrenal, abdominal paraspinal, and mediastinal [11].

Clinical presentation of adult neuroblastoma is variable; the most common symptoms are abdominal pain and sequelae from impingement on neighbouring viscera. However, neuroblastoma may also present as adrenal incidentalomas and may secrete catecholamines, aldosterone or adrenocorticotropic hormone (ACTH) [1]. Medications may also increase plasma metanephrine levels [12], including venlafaxine taken by our patient, which has been reported to increase normetanephrine to more than four-fold the upper limit of normal [13–15]. Isolated elevations in metanephrine or normetaphrine levels at least three-fold the upper limit of normal, however, are unlikely to be false positives. This should prompt further investigations to detect a PPGL or neuroblastoma [16].

Neuroblastomas may be misdiagnosed as phaeochromocytomas given the ability to secrete catecholamines. They may also have similar radiological features, including heterogeneous contrast uptake on CT and poorly-defined margins [17]. They are usually identified in the retroperitoneum or pelvis [17]. To date, all reported cases of catecholamine-secreting adrenal adult neuroblastomas have been very large, ranging in size from 8 cm to 24.1 cm. The resected neuroblastoma described in this case was 15 cm in diameter (Fig. 2).

Interestingly, the neuroblastoma described here had biochemical and imaging profiles suggestive of a pseudohypoxic subtype phaeochromocytoma; there were elevated normetanephrine and 3-MT levels but normal metanephrine levels, together with both FDG and SSTR-avidity on nuclear imaging.

Surgery is the primary treatment for localised disease, with adjuvant chemotherapy and radiotherapy offered depending on stage, histology, grade and genetic prognostic factors [1]. A high proportion of adult patients – up to 75% of cases in one series [11] – present with metastatic disease. Thus, there is an imperative for genomic testing in these patients to evaluate for targeted systemic treatment options.

The genomic landscape of adult neuroblastoma differs from paediatric tumours. For example, a published cohort analysis of 26 adult neuroblastoma patients who had varying methods of genomic testing describes 42% with activating ALK mutations and 58% with ATRX loss-of-function mutations [11]. Both of these mutations occur less frequently in paediatric tumours, which are more commonly driven by MYCN-amplification [11, 18]. The high frequency of ALK-mutations in adult neuroblastoma offers the potential for targeted therapy with ALK-inhibitors for patients with metastases or recurrent disease, as is available for our patient.

There may be additional mutations in adults as yet unrecognised due to rarity of the condition and limited available tumour genomic data. Further research is required to investigate genomic differences between adult and paediatric cases, and understand why adult neuroblastomas carry a worse prognosis than paediatric neuroblastomas [11].

Including this case, only seven cases of catecholamine-secreting adrenal neuroblastoma in adults have been described in the literature [1–7]. Prior to surgical resection and histological review, the differentials for this case included pseudohypoxic subtype phaeochromocytoma, and composite phaeochromocytoma. There are no established clinical or radiological criteria to distinguish catecholamine-secreting neuroblastoma from PPGL. Histology was critical for this case given adjuvant therapy for neuroblastoma differs from PPGL, with the former treated with radiotherapy. Surveillance and genetic testing also differ between the two pathologies. Diagnosing ALK-positive neuroblastoma triggers consideration of ALK-targeted therapy, which is not relevant for PPGL. A critical eye is required for the accurate diagnosis and management of malignant adrenal incidentalomas.

## Data Availability

The data for this case report are available upon request from the corresponding author, given that ethics clearance has been obtained.

## Abbreviations

3-MT: 3-Methoxytyramine
ACTH: Adrenocorticotropic hormone
ALK: Anaplastic lymphoma kinase
ATRX: Alpha-thalassemia mental retardation X-linked
CT: Computed tomography
Ga-TATE: [^68^ Ga]Ga-DOTATATE
MYCN: V-myc myelocytomatosis viral-related oncogene, neuroblastoma derived
PPGL: Phaeochromocytoma and paraganglioma
PET: Positron emission tomography
SSTR: Somatostatin receptor

## References

[1] Telecan T, Andras I, Bungardean MR, et al. Adrenal gland primary neuroblastoma in an adult patient: A case report and literature review. Medicina. 2023;59(1):33. 10.3390/medicina59010033 PMC9860607 36676657

[2] Suzuki H, Honzumi M, Funada M, et al. Metachronous bilateral adrenal neuroblastoma. Cancer. 1985;56:1490–2. 4027884 10.1002/1097-0142(19850915)56:6<1490::aid-cncr2820560645>3.0.co;2-k

[3] Genc H, Haciyanli M, Haciyanli SG, et al. An adult adrenal neuroblastoma: A case report. Acta Chir Belg. 2005;105(6):673–6. 16438086 10.1080/00015458.2005.11679803

[4] Schalk E, Mohren M, Koenigsmann M, et al. Metastatic adrenal neuroblastoma in an adult. Onkologie. 2005;28(6–7):353–5. 15933424 10.1159/000085526

[5] Manjunath S, Mouli S, Singh KR, et al. Neuroblastoma in late adolescence: Case report and review of literature. World J Endoc Surg. 2018;10(3):157–62.

[6] McCarthy LC, Chastain K, Flatt TG, et al. Neuroblastoma in adolescents and children older than 10 years: Unusual clinicopathologic and biologic features. J Pediatr Hematol Oncol. 2019;41:586–95. 30973487 10.1097/MPH.0000000000001485

[7] Guzman Gómez GE, Urbano MA, Martínez V. Adrenal neuroblastoma producing catecholamines diagnosed in adults: Case report. Case Rep Oncol. 2022;15:682–6. 36157691 10.1159/000520125 PMC9386402

[8] Sherlock M, Scarsbrook A, Abbas A, et al. Adrenal incidentaloma. Endocr Rev. 2020;41(6):775–820. 32266384 10.1210/endrev/bnaa008 PMC7431180

[9] Nieman LK. Approach to the patient with an adrenal incidentaloma. J Clin Endocrinol Metab. 2010;95(9):4106–13. 20823463 10.1210/jc.2010-0457 PMC2936073

[10] Yilmaz N, Avsar E, Tazegul G, et al. Clinical characteristics and follow-up results of adrenal incidentaloma. Exp Clin Endocrinol Diabetes. 2021;129(05):349–56. 31958848 10.1055/a-1079-4915

[11] Suzuki M, Kushner BH, Kramer K, et al. Treatment and outcome of adult-onset neuroblastoma. Int J Cancer. 2018;143(5):1249–58. 29574715 10.1002/ijc.31399 PMC6103828

[12] Eisenhofer G, Pamporaki C, Lenders JW. Biochemical assessment of pheochromocytoma and paraganglioma. Endocr Rev. 2023;44(5):862–909. 36996131 10.1210/endrev/bnad011

[13] Neary NM, King KS, Pacak K. Drugs and pheochromocytoma—don’t be fooled by every elevated metanephrine. N Engl J Med. 2011;364(23):2268–70. 21651412 10.1056/NEJMc1101502#SA1 PMC4724800

[14] Mañas-Martínez AB, Aragoneses-Calvo A, Matei A, et al. Venlafaxine drug interaction in the diagnosis of pheochromocytoma. Endocrinologia y Nutricion: Organo de la Sociedad Espanola de Endocrinologia y Nutricion. 2016;63(10):569–70. 27751751 10.1016/j.endonu.2016.08.006

[15] Remde H, Pamporaki C, Quinkler M, et al. Improved diagnostic accuracy of clonidine suppression testing using an age-related cutoff for plasma normetanephrine. Hypertension. 2022;79(6):1257–64. 35378989 10.1161/HYPERTENSIONAHA.122.19019

[16] Lenders JW, Duh Q-Y, Eisenhofer G, et al. Pheochromocytoma and paraganglioma: an endocrine society clinical practice guideline. J Clin Endocrinol Metab. 2014;99(6):1915–42. 24893135 10.1210/jc.2014-1498

[17] Tateishi U, Hasegawa T, Makimoto A, et al. Adult neuroblastoma: Radiologic and clinicopathologic features. J Comput Assist Tomogr. 2003;27(3):321–6. 12794593 10.1097/00004728-200305000-00004

[18] Sarnacki S, Pio L. Neuroblastoma: Clinical and surgical management. Cham (Switzerland): Springer Nature; 2020.

